# Hypoxic stabilization of mRNA is HIF-independent but requires mtROS

**DOI:** 10.1186/s11658-018-0112-2

**Published:** 2018-10-04

**Authors:** Grey W Fortenbery, Brinda Sarathy, Kristen R Carraway, Kyle D Mansfield

**Affiliations:** 1 Brody School of Medicine, East Carolina University, Greenville, NC 27834 USA; 2 Biochemistry and Molecular Biology, Brody School of Medicine, East Carolina University, Greenville, NC 27834 USA

**Keywords:** Hypoxia, Hypoglycemia, HIF, Mitochondrial reactive oxygen species, mRNA stability

## Abstract

**Background:**

Tissue ischemia can arise in response to numerous physiologic and pathologic conditions. The cellular response to decreased perfusion, most notably a decrease in glucose and oxygen, is important for cellular survival. In response to oxygen deprivation or hypoxia, one of the key response elements is hypoxia inducible factor (HIF) and a key protein induced by hypoxia is vascular endothelial growth factor (VEGF). Under hypoxia, we and others have reported an increase in the half-life of VEGF and other hypoxia related mRNAs including MYC and CYR61; however, the mediator of this response has yet to be identified. For this study, we sought to determine if HIF-mediated transcriptional activity is involved in the mRNA stabilization induced by hypoxia.

**Methods:**

HEK293T or C6 cells were cultured in either normoxic or hypoxic (1% oxygen) conditions in the presence of 1 g/L glucose for all experiments. Pharmacological treatments were used to mimic hypoxia (desferroxamine, dimethyloxaloglutamate, CoCl_2_), inhibit mitochondrial respiration (rotenone, myxothiazol), scavenge reactive oxygen species (ROS; ebselen), or generate mitochondrial ROS (antimycin A). siRNAs were used to knock down components of the HIF transcriptional apparatus. mRNA half-life was determined via actinomycin D decay and real time PCR and western blotting was used to determine mRNA and protein levels respectively.

**Results:**

Treatment of HEK293T or C6 cells with hypoxic mimetics, desferroxamine, dimethyloxaloglutamate, or CoCl_2_ showed similar induction of HIF compared to hypoxia treatment, however, in contrast to hypoxia, the mimetics caused no significant increase in VEGF, MYC or CYR61 mRNA half-life. Knockdown of HIF-alpha or ARNT via siRNA also had no effect on hypoxic mRNA stabilization. Interestingly, treatment of HEK293T cells with the mitochondrial inhibitors rotenone and myxothiazol, or the glutathione peroxidase mimetic ebselen did prevent the hypoxic stabilization of VEGF, MYC, and CYR61, suggesting a role for mtROS in the process. Additionally, treatment with antimycin A, which has been shown to generate mtROS, was able to drive the normoxic stabilization of these mRNAs.

**Conclusion:**

Overall these data suggest that hypoxic mRNA stabilization is independent of HIF transcriptional activity but requires mtROS.

## Background

Tissue ischemia is implicated in numerous pathologic conditions such as myocardial infarction, stroke, tumor angiogenesis, and wound healing. Although ischemia results in the deprivation of numerous nutrients, the deprivation of oxygen and glucose arguably have the greatest impact on cellular metabolism, and as such, appropriate adaptation is critical to cellular survival during an ischemic event [1, 2]. In response to ischemia, multiple signaling pathways and transcription factors are activated to mitigate the detrimental effects of ischemia on the cell leading to increased cell survival [2–4].

The cellular response to hypoxia has been well studied and much is known about the transcriptional response that is initiated upon exposure to low oxygen conditions. The main mediators of this response are the Hypoxia Inducible Factor (HIF) family of transcription factors, with the oxygen-dependent HIF-specific prolyl hydroxylases (PHDs) playing a critical role in the oxygen dependent regulation of the HIF-α subunits [5–7]. Under normoxic conditions (21% O_2_) the HIF-α subunit is hydroxylated on two key prolines within the oxygen dependent degradation domain (ODD). The hydroxylated ODD is then recognized by von Hippel-Lindau (VHL), a component of an E3 ubiquitin ligase complex, which targets the HIF-α protein for degradation by the 26S proteasome [8–10]. When cells are oxygen deprived, hydroxylation does not occur, HIF-α is stabilized and translocates into the nucleus. There, along with its heterologous binding partner Aryl Hydrocarbon Receptor Nuclear Translocator (ARNT, HIF-β), it then binds Hypoxia Response Elements (HREs) within promoters and activates transcription of target genes, such as VEGF and GLUT1, that are necessary for adaptation to low oxygen levels.

While the HIF hydroxylases play a key role in oxygen sensing and signaling, additional signaling pathways also affect HIF-dependent and independent hypoxic responses. For example, we and others have shown that the mitochondrion, an organelle that has evolved specifically for oxygen use, also plays a critical role in cellular oxygen sensing [11–16]. Inhibition of mitochondrial electron transport by pharmacological agents or genetic ablation inhibits cellular oxygen sensing and prevents HIF-α protein stabilization in response to low oxygen levels [11–15]. This has been shown to be due to a reduction in mitochondrial reactive oxygen species (mtROS) production that is both necessary and sufficient for the inhibition of HIF prolyl hydroxylase activity [7, 15, 16]. mtROS have also been shown to be generated in response to cardiomyocyte ischemia [17], and have also been shown to impact a number of HIF-independent cellular processes, including myocardiocyte contraction [18, 19], IL-6 production [20], and glutathione reduction [21].

Although the transcriptional response to hypoxia has been well-established, the post-transcriptional response is not completely understood and virtually unexplored in the context of ischemic disease [22, 23]. One such post-transcriptional change that is seen under hypoxia is an increased mRNA stability for certain hypoxia-related mRNAs, including VEGF, GLUT1, MYC and CYR61 [24–26]. Physiologically, changes in mRNA stability do not have a direct effect on cellular function, but can significantly alter mRNA steady state levels, which in turn ultimately affects the amount of protein product produced [27].

The posttranscriptional regulation of VEGF mRNA in particular has been studied in multiple cell types [24, 28–32], and is thought to involve AU Rich Elements (AREs) and the RNA binding proteins (RBPs) HuR, PTB, hnRNPL, and others [24, 28, 33–36]. The signaling events preceding VEGF mRNA stabilization by hypoxia have also been studied, with stress activated protein kinases playing an essential role [24, 28, 37].We have recently expanded upon the VEGF studies and show that oxygen and glucose deprivation (hypoxia/hypoglycemia) synergize to induce the stabilization of a number of hypoxia-related mRNAs including VEGF, MYC, and CYR61 [25]. In addition, we have also uncovered a potential role for mRNA modification N6-methyladenosine in this process [26], but the regulatory proteins and signaling pathways involved in the global stabilization of mRNA in response to hypoxia/hypoglycemia are still unknown. In addition, there have been no investigations of the oxygen-sensing pathways involved or whether the HIF transcriptional response is required for the hypoxic stabilization of mRNA.

Therefore, we set out to investigate the role that HIF transcriptional activity plays in the stabilization of mRNA under hypoxic/hypoglycemic conditions. Overall, our results suggest that the hypoxic/hypoglycemic stabilization of mRNA does not require the HIF-mediated transcriptional response but that mtROS is both necessary for the response and sufficient to substitute for hypoxia. Overall, these results provide additional information into the mechanism of hypoxic/hypoglycemic mRNA stabilization.

## Methods

### Cell lines

HEK293T and C6 cells were obtained from ATCC and maintained in high glucose (4 g/L) DMEM (Corning) supplemented with 10% FBS, 2 mM Glutamine, and 1X Pen/Strep and passaged when approximately 85–90% confluent. For experiments, cells were plated at 0.5 × 10^6^ cell per well of a 6 well plate (CytoOne) in high glucose (4.0 g/L) media and allowed to attach/recover for 18–24 h. The next day, the media was removed and replaced with 1 g/L glucose DMEM and if indicated, treated with the appropriate pharmacological agent, which were all obtained from Sigma-Aldrich. Hypoxic treatments were carried out in a Ruskin In Vivo 400 Hypoxia Hood (Baker) maintained at 37 °C, 5% CO_2_, 70% humidity and 1% oxygen. All cell lysis/extractions of hypoxic samples were done in the hypoxic workstation to avoid reoxygenation.

### RNA extraction

Trizol (Life Technologies) was used for all RNA extractions according to the manufacturer’s protocol. RNA purity and quantity was determined via NanoDrop 1000 (Thermo Scientific).

### PCR

Reverse transcription was performed on 1 μg of total RNA in a 20 μl reaction with the iScript cDNA synthesis kit (Bio-Rad). Quantitative real-time PCR was performed using a Roche Lightcycler 96 with Fast Start Essential DNA Green (Roche) and primers from IDT. Primer efficiency was verified to be over 95% for all primer sets used. Quantification of mRNA was carried out via ΔΔCT analysis using GAPDH mRNA and the respective control condition for normalization. All real-time PCR primer sets were designed so the products would span at least one intron (> 1 kb when possible), and amplification of a single product was confirmed by melting curve analysis. Primers used were as follows:

Human Primers used for HEK293T Real Time PCR:

**Table:** 

Ta rget	Gene Symbol	Forward Primer	Reverse Primer
ARNT1	ARNT	TCGTGAGCAGCTTTCCACTTCAGA	ACAGAGCTACTGCCACACCTCATT
ARNT2	ARNT2	GCCCTGTGAAAGAAGGAGAA	ATCAGCGTCTTCTTCAGGTATG
CYR61	CYR61	CTTCTCCACTTGACCAGGCT	AGTCCTCGTTGAGCTGCTTG
GAPDH	GAPDH	AAGGTCGGAGTCAACGGATTTGGT	AGCCTTGACGGTGCCATGGAATTT
GLUT1	SLC2A1	TATCGTCAACACGGCCTTCACTGT	CACAAAGCCAAAGATGGCCACGAT
HIF1	HIF1A	CCGAATTGATGGGATATGAGCCAG	TTGGCAAGCATCCTGTACTGTCCT
HIF2	EPAS1	AAGCTGAAGCGACAGCTGGAGTAT	GTACATTTGCGCTCAGTGGCTTGT
MYC	MYC	TCCTCGGATTCTCTGCTCTCCT	AGAAGGTGATCCAGACTCTGACCT
VEGF	VEGFA	ATCTTCAAGCCATCCTGTGTGC	CAAGGCCCACAGGGATTTTC

Rat Primers used for C6 Real Time PCR:

**Table:** 

Target	Gene Symbol	Forward Primer	Reverse Primer
VEGF	VEGFA	ATCTTCAAGCCGTCCTGTGTGC	CAAGGCTCACAGTGATTTTCTGG
MYC	MYC	AGCTCCTCGCGTTATTTGAAGCCT	AGATGAAATAGGGCTGCACCGAGT
MDM2	MDM2	CTCAGAAGATTACAGCCTGAGTG	CTGATAGACTGTGACCCGATAGA
GAPDH	GAPDH	CGTCTCATAGACAAGATGGTGAA	CGTTGATGGCAACAATGTCC

### mRNA decay rates

mRNA levels were determined by real-time quantitative PCR at 0, 1, or 2, hours after the addition of 5 μg/ml actinomycin D. GAPDH mRNA and time 0, untreated controls were used for ΔΔCT normalization. Half-lives were determined by regression of the semi-logarithmic concentration versus time data using an Excel Half-Life add-in, *PK Functions* [38]. All half-lives > 6 h or those calculated to have a negative half-life (infinitely stable) were converted to 6 h which was the maximum half-life reliably calculated by this method [25].

### Western blots

Whole cell lysates were prepared in whole cell extract buffer (50 mM Tris pH 7.4, 150 mM NaCl, 5 mM EDTA, 0.1% SDS, and complete protease inhibitor (Promega)). Equal amounts of protein (30–50 μg) were electrophoresed on a mini protean any KD acrylamide gel (BioRad), transferred to Hybond ECL nitrocellulose (GE Healthcare). Transfer was verified via Ponceau S staining then blot was blocked with 5% nonfat dry milk in TBST for 1 hour at room temperature, followed by primary antibody overnight at 4 °C. After washing extensively, blots were incubated for 1–2 h at room temperature with appropriate HRP-linked secondary antibody (GE Healthcare), washed, developed using Pierce ECL Western Blotting Substrate, and exposed to film for detection. Primary Antibodies used and their concentrations were as follows:

**Table:** 

Antibody	Catalog #	Vendor	Dilution
ARNT1	SC-17811	Santa Cruz	1:500
ARNT2	SC-393683	Santa Cruz	1:500
GAPDH	SC-365062	Santa Cruz	1:1000
GLUT1	PA5–16793	Thermo Fisher	1:250
HIF-1α	3716	Cell Signalling	1:1000 (in BSA)
HIF-2α	NB100–122	Novus	1:1000
LDH	SC-133123	Santa Cruz	1:1000
Tubulin	MA1–850	Thermo Fisher	1:1000

### siRNA transfections

siRNAs were transfected using Lipofectamine 2000 (Life Technologies) per manufacturer’s protocol using 100 pM siRNAs/well of a 6 well plate. siRNAs used in this study were as follows:

**Table:** 

siRNA	ID #	Vendor
Negative Control	AM4635	Ambion
ARNT1	S1613	Ambion
ARNT2	Hs.Ri.ARNT2.13.2	IDT
HIF1	S102664053	Qiagen
HIF2	S102663038	Qiagen

### Statistical analysis

All experiments were performed on at least 3 separate occasions to generate biological replicates. qPCR was performed at least twice on each sample for technical replicates. Half-lives were calculated for each biological replicate and then averaged together to determine final value and standard deviation of experiment. Statistical significance was calculated by two-tailed paired Student’s t-test comparing experimental to control conditions. A *P*-value less than or equal to 0.05 was defined as statistically significant and less than or equal to 0.1 was considered reportable.

## Results

### Activation of the HIF pathway is not sufficient to stabilize mRNA

In our previous report, we showed that high glucose (4 g/L) could prevent the hypoxic stabilization of many mRNAs including VEGF, MYC, and CYR61 in both HEK293T and rat C6 cells [25]. Interestingly, the HIF-mediated transcriptional increase in mRNA levels has also been reported to be attenuated by high glucose levels as well [39–41] suggesting that the two responses may be linked.

**Fig. 1:**
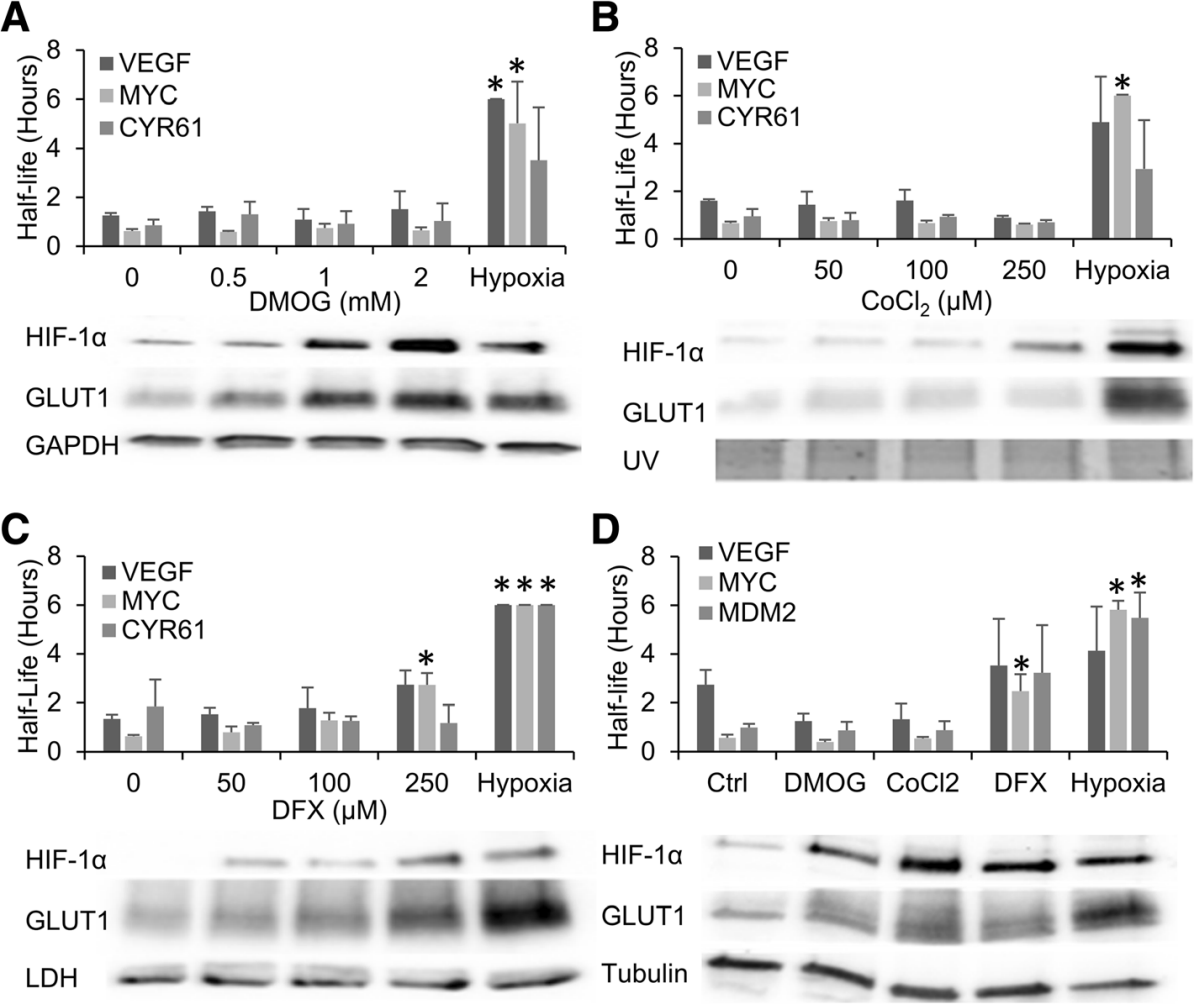
mRNA stability is induced by hypoxic exposure but not treatment with hypoxic mimetics. HEK293T (**a-c**) or C6 (**d**) cells exposed to 24 h of Normoxia and either 1% Oxygen (Hypoxia) or the indicated concentrations of DMOG (**a**), CoCl_2_ (**b**), or DFX (**c**) in the presence 1 g/L glucose before being treated with actinomycin D for 0, 1 or 2 h. mRNA levels of VEFG, MYC, and CYR61 were determined via qPCR, normalized to GAPDH and 0 h time point and half-lives determined. Protein was analyzed via western blotting to confirm treatments had desired effect of HIF induction. Data represents average of *N* = 3 (HEK293T) or 4 (C6) ± SD for each condition. *P ≤ 0.05 from respective Normoxic (0, Ctrl) condition

To investigate whether induction of the HIF pathway is involved in the mRNA stabilization response, we used the hypoxic mimetics desferroxamine (DFX), dimethyloxalylglycine, (DMOG), and cobalt chloride (CoCl_2_) to induce stabilization of HIF-α protein independent of the oxygen concentrations. HEK293T cells were treated for 24 h in the presence of low glucose (1 g/l) with either increasing doses of DMOG, DFX, or CoCl_2_ in normoxic conditions, or exposed to 1% oxygen (hypoxia). mRNA half-life was then determined via actinomycin D decay and protein isolated for western blotting. As previously reported [25], exposure to hypoxia in a low glucose environment resulted in a robust and significant stabilization of VEGF, MYC, and CYR61 mRNA and resulted in a moderate level of HIF-1α protein stabilization and an increase in GLUT1(a known HIF transcriptional target) protein levels as compared to the loading control (Fig. 1a-c). Treatment of the cells with DMOG resulted in an extremely robust stabilization of HIF-1α protein in a dose dependent manner with the 1 mM dose giving a similar response to hypoxia and the 2 mM dose giving a slightly more robust response than the hypoxic control (Fig. 1a). However, despite the robust HIF response, there was no significant increase in the VEGF, MYC, or CYR61 mRNA half-lives at any dose of DMOG (Fig. 1a).

Similarly, treatment of the cells with the iron chelator DFX also saw stabilization of HIF-1α protein with 250 μM of DFX resulting in similar stabilization of HIF-1α protein as seen in hypoxia (Fig. 1c). Again, there was no stabilization of VEGF or CYR61 mRNA at any dose of DFX; however, there was significant stabilization of MYC mRNA seen at 250 μM of DFX (Fig. 1c) although it was still less than the hypoxic response. Treatment with CoCl_2_ also resulted in moderate HIF-1α stabilization similar to the hypoxic condition seen at 250 μM (Fig. 1b) but failed to elicit any significant increase in mRNA half-life.

Similar results were seen with C6 cells where 2 mM DMOG, 250 μM CoCl_2_, or 250 μM DFX elicited HIF-1α protein stabilization and GLUT-1 induction similar to the hypoxic treatment, with both DMOG and CoCl_2_ failing to significantly increase the mRNA half-life of VEGF, MYC, or MDM2. Interestingly, DFX did induce an increase in mRNA half-life of all three mRNAs tested. However, while the highest dose of DFX did elicit a response with significant stabilization of MYC in both cell types, the extent of mRNA stabilization from any of the three mimetic treatments did not correlate with the level of HIF-1α protein stabilization seen with those treatments. This suggests that HIF-α stabilization is not sufficient to increase mRNA half-life on its own and that the DFX is likely working through some alternative mechanism.

### Hypoxic mRNA stability is not dependent on HIF transcriptional activity

**Fig. 2:**
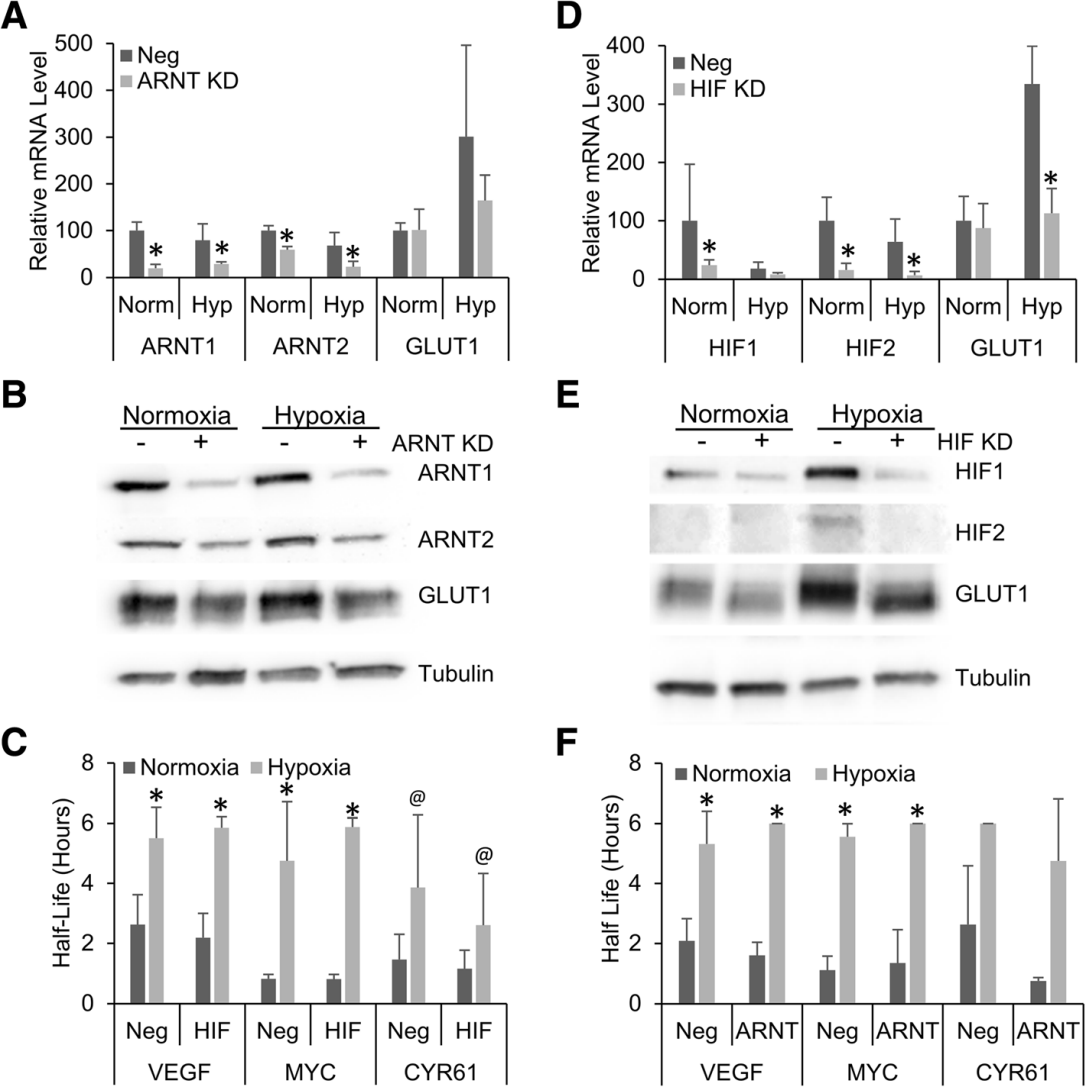
Hypoxic mRNA stability does not require HIF transcriptional activity. HEK293T cells were simultaneously transfected with siRNAs targeting ARNT1 and 2 (ARNT KD), HIF-1α and 2α (HIF KD) or negative control siRNA (Neg) for 48 h before being exposed to 24 h of Normoxia or Hypoxia (1% O_2_) in the presence of 1 g/L glucose. Cells were treated with actinomycin D for 0, 1 or 2 h. RT-PCR (**a, d**) and western blotting (**b, e**) of 0 h time point were used to assess effectiveness of knockdown. **c, f** mRNA levels of VEFG, MYC and CYR61 were determined via qPCR, normalized to GAPDH and 0 h time point and half-lives determined. Data represents average of N = 3 ± SD for each condition. *P ≤ 0.05; @*P* ≤ 0.1 from respective control

While HIF induction alone was not sufficient to induce the mRNA stabilization response, we next examined if HIF transcriptional activity was necessary for the hypoxic response. To test this, HEK293T cells were simultaneously transfected with siRNAs for ARNT1 and ARNT2, allowed to recover for 48 h, and then transferred to 1 g/L glucose media before being exposed to normoxia or hypoxia for 24 h. As before, mRNA half-life was determined via actinomycin D decay and protein isolated for western blotting. Following siRNA knockdown, there was a significant decrease in ARNT1 and ARNT2 mRNA levels in the cell and a moderate decrease in the hypoxic levels of GLUT1 mRNA, a known HIF transcriptional target (Fig. 2a). Correlated with this knockdown in RNA levels, there was also a dramatic decrease in ARNT and GLUT1 protein in the cells transfected with siRNAs under both normoxic and hypoxic conditions compared to controls (Fig. 2b). Interestingly, when exposed to hypoxia the ARNT knock-down cells had a similar mRNA stabilization response as the control cells (Fig. 2c). This suggests that ARNT is not necessary for the increase in mRNA half-life seen under hypoxia.

Next, we investigated if directly knocking down the HIF-α subunits had a similar effect. As before, HEK293T cells were simultaneously transfected with siRNAs for HIF-1α and HIF-2α and allowed to recover for 48 h. These cells were then transferred to 1 g/L glucose media and subsequently exposed to normoxia or hypoxia as previously described. Levels of HIF mRNA in both control cells and transfected cells were determined by RT-PCR and cell protein was isolated and analyzed via Western blotting. As was expected, the siRNAs effectively knocked down both HIF mRNA and protein levels and significantly inhibited GLUT1 transcription and subsequent protein production. (Fig. 2d and e). Furthermore, when exposed to hypoxia, the cells treated with HIF siRNAs showed similar stabilization of VEGF, MYC, and CYR61 mRNA as the negative siRNA controls (Fig. 2f). These results again suggest that when HIF transcriptional activity is blocked it has no effect on the hypoxic stabilization of mRNA, indicating that HIF is not required for the observed response.

### Functional mitochondria are required for hypoxic mRNA stabilization

**Fig. 3:**
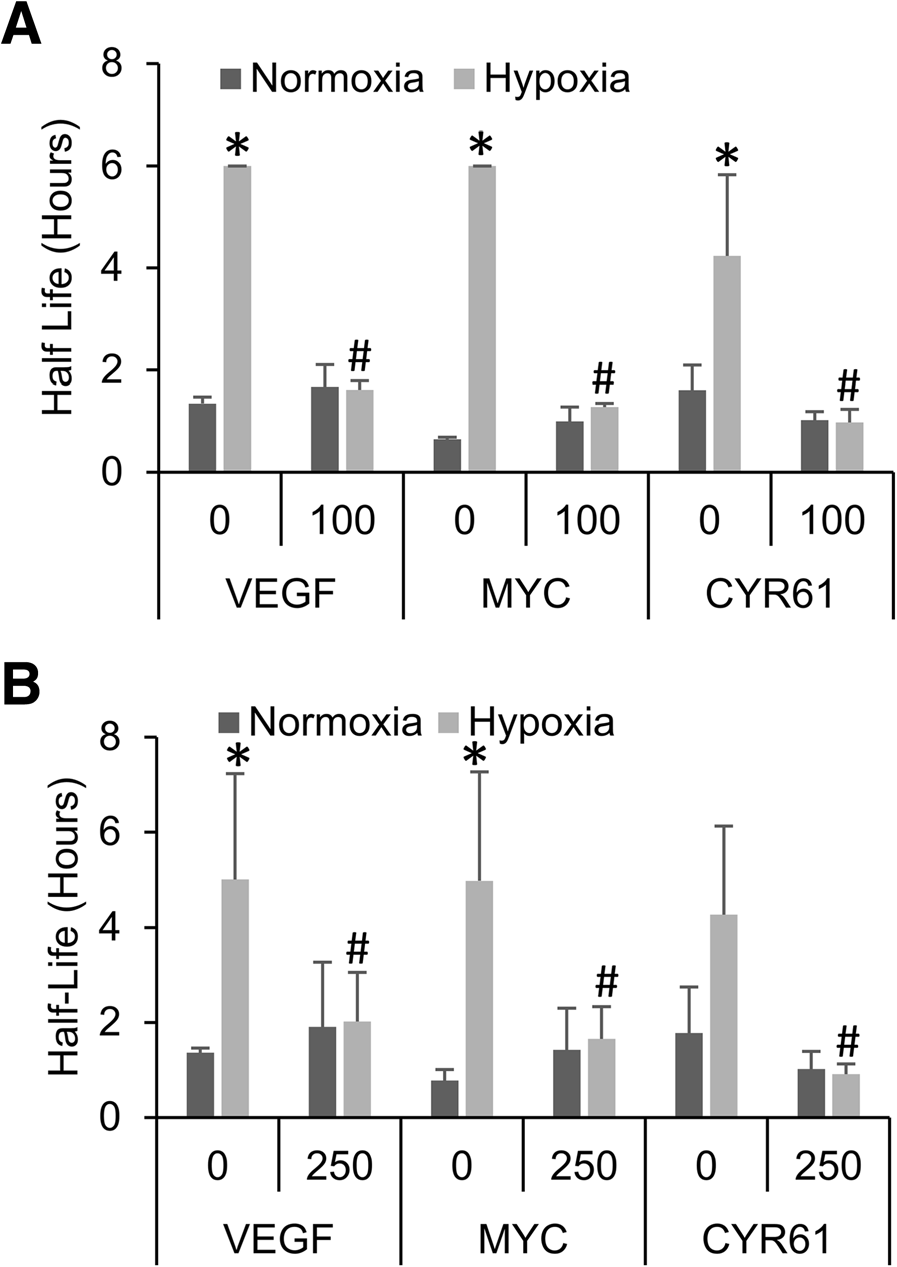
Hypoxic mRNA stability requires functional mitochondria. HEK293T cells pre-treated with mitochondrial inhibitors rotenone (**a**) or myxothiazol (**b**), and then exposed to 24 h of Normoxia or Hypoxia (1% O_2_) in the presence of 1 g/L glucose before being treated with actinomycin D for 0, 1 or 2 h. mRNA levels of VEGF, MYC and CYR61 were determined via qPCR, normalized to GAPDH and 0 h time point and half-lives determined. Data represents average of *N* ≥ 3 ± SD for each condition. *P ≤ 0.05 from respective Normoxic condition; #*P* ≤ 0.05 from vehicle treated (0) hypoxic sample

We next tested the involvement of the mitochondria in the sensing and signaling of hypoxia/hypoglycemia as we and others have shown it to be involved in other HIF-dependent and independent responses [19–21, 42]. Once again, HEK293T cells were switched to 1 g/l glucose media and then pre-treated for 15 min with either the complex I inhibitor rotenone or the complex III Q_o_ site inhibitor myxothiazol, before being exposed to hypoxia (1% O_2_) for 24 h and half-lives determined via actinomycin D decay. Treatment with either rotenone (Fig. 3a) or myxothiazol (Fig. 3b) completely blocked the hypoxic stabilization of VEGF, MYC and CYR61 mRNA suggesting that the mitochondria are playing a role in the sensing and/or signaling of the hypoxic condition.

### Hypoxic mRNA stabilization is mediated by mtROS

**Fig. 4:**
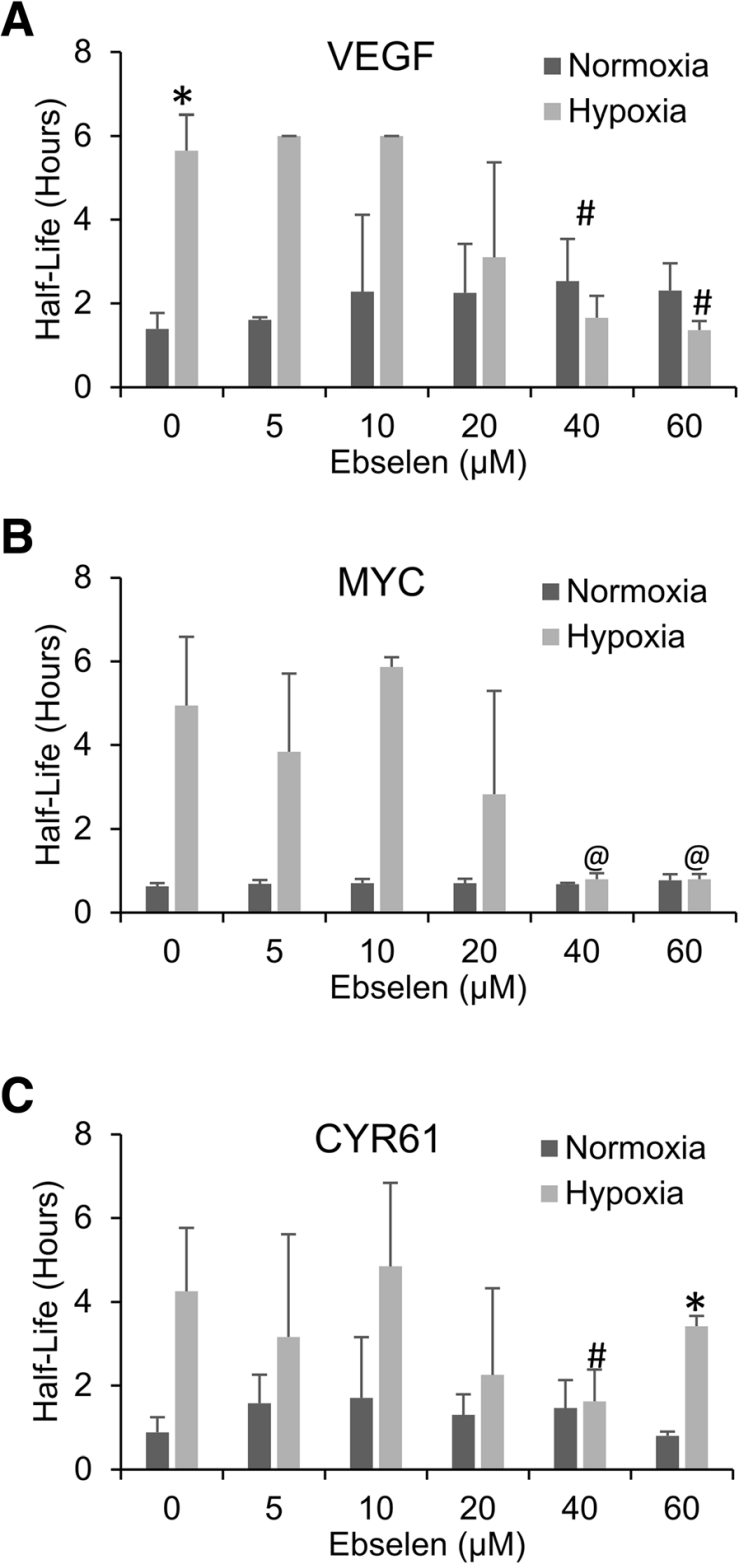
ROS is necessary for hypoxic mRNA stabilization. HEK293T cells pre-treated with indicated doses of glutathione peroxidase mimetic ebselen and then exposed to 24 h of Normoxia or Hypoxia (1% O_2_) in the presence of 1 g/L or 4 g/L glucose before being treated with actinomycin D for 0, 1 or 2 h. mRNA levels of VEGF (**a**), MYC (**b**) and CYR61 (**c**) were determined via qPCR, normalized to GAPDH and 0 h time point and half-lives determined. Data represents average of N = 3 ± SD for each condition. *P ≤ 0.05 from respective normoxic control condition; #*P* ≤ 0.05; @*p* ≤ 0.1 from vehicle treated (0) hypoxic sample

To investigate the role of mtROS in the hypoxic stabilization of mRNA, we first treated cells with the glutathione peroxidase mimetic ebselen which has been shown to efficiently reduce hypoxic mtROS levels and prevent other hypoxic responses [11, 14]. HEK293T cells were switched to 1 g/L glucose media, pretreated for 30 mins with increasing doses of ebselen, and then exposed to 24 h of hypoxia before half-lives were determined via actinomycin D decay. Treatment with ebselen inhibited the hypoxic stabilization of both VEGF and MYC at all doses (Fig. 4a, b), while CYR61 mRNA stabilization was inhibited only with the lower two doses (Fig. 4c).

**Fig. 5:**
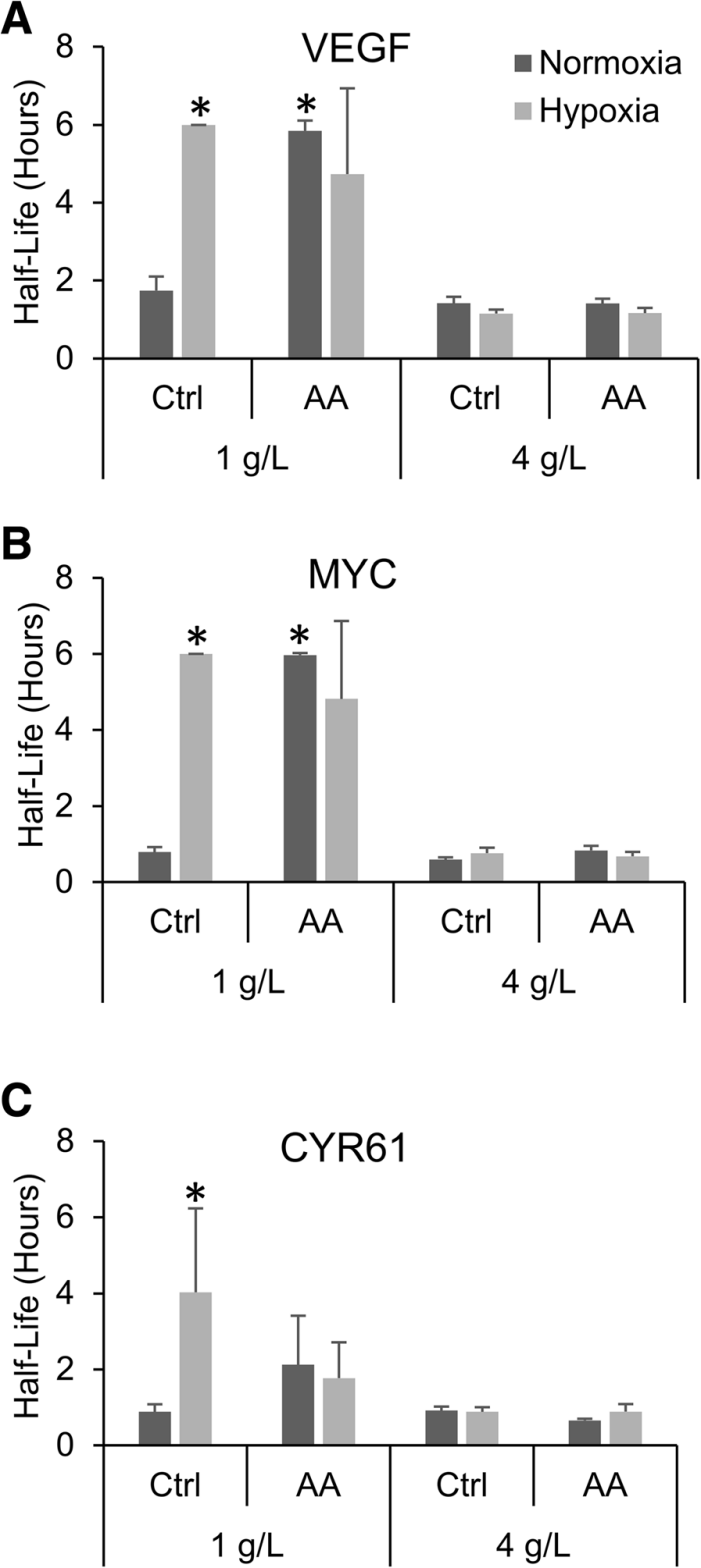
mtROS production is sufficient for mRNA stabilization. HEK293T cells pre-treated with 10 μM of the Complex III Qi site inhibitor antimycin A and then exposed to 24 h of Normoxia or Hypoxia (1% O_2_) in the presence of 1 g/L or 4 g/L glucose before being treated with actinomycin D for 0, 1 or 2 h. mRNA levels of VEGF (**a**), MYC (**b**) and CYR61 (**c**) were determined via qPCR, normalized to GAPDH and 0 h time point and half-lives determined. Data represents average of *N* = 3 ± SD for each condition. *P ≤ 0.05 from 1 g/L normoxic control condition

Given that mtROS appeared to be necessary for the hypoxic mRNA stabilization we also wanted to investigate if generation of mtROS was sufficient to induce the response independent of hypoxia. To induce generation of mtROS independent of hypoxia, HEK293T cells were treated with 10 μM of the mitochondrial Complex III Qi site inhibitor, antimycin A which has been shown to generate mtROS and mimic the hypoxic response [43–45]. As predicted, when HEK293T cells were cultured in 1 g/L glucose media, treatment with antimycin A under normoxic conditions induced the mRNA stabilization of both VEGF and MYC to similar levels seen with hypoxic treatment of the control cells while not affecting the hypoxic response of the treated cells (Fig. 5a and b). Antimycin A treatment also increased CYR61 mRNA half-life but to a lesser degree than hypoxia (Fig. 5c). Furthermore, as previously reported [25], when the cells were cultured in 4 g/L glucose media, the hypoxic mRNA stabilization response was inhibited (Fig. 5a-c). Interestingly, under these same high glucose conditions, antimycin A treatment also fails to increase the mRNA half-lives of VEGF, MYC and CYR61 suggesting that induction of mtROS can substitute for hypoxia but not hypoglycemia.

## Discussion

The response to hypoxia involves many different changes in gene expression [22]. The transcriptional changes that occur through the actions of the Hypoxia Inducible Factor family of transcription factors have been well studied, however, there are also many posttranscriptional events that likely play a role as well [23, 46]. However, the contribution of the transcriptional response to the posttranscriptional response has not been well studied.

One well studied posttranscriptional response to hypoxia is the observed stabilization of a subset of mRNAs, that are important for the adaptive response. The stabilization of VEGF mRNA in particular has been examined in great detail and has been shown to involve MAP kinase signaling pathways and multiple RNA binding proteins, including HuR [24, 28, 30, 37]. Interestingly, in contrast to our results, the RNA binding complex in those studies did appear to be under the control of the HIF pathway as knockout of VHL induced constitutive complex binding to both VEGF and GLUT1 3’ UTR and resulted in increased mRNA half-life similar to hypoxia [29, 47].

While we didn’t investigate the role of VHL in our system, treatment with the hypoxic mimetics DMOG, DFX, and CoCl_2_ should theoretically have the same effect of stabilizing HIF-α subunits and inducing the transcriptional response. However, our data utilizing the hypoxic mimetics DFX, DMOG, and CoCl_2_ suggests that HIF protein stabilization is not sufficient to drive the mRNA stabilization of VEGF and MYC seen under hypoxia. While the highest dose of DFX did moderately increase MYC mRNA half-life, treatment with any of the three mimetics had no significant effect on VEGF or CYR61 mRNA half-life despite its ability to stabilize HIF-1α similar to hypoxia, suggesting that HIF-stabilization alone is not sufficient to induce the response. These data are supported by a recent publication showing that CoCl_2_ treatment increased VEGF protein translation but had no effect on its mRNA stabilization [48]. It is possible that the difference in the results lie in the fact that VHL null cells have experienced increased HIF-α long-term, while the mimetics studies were only carried out for 24 h.

Although HIF does not seem to be involved in the posttranscriptional response, other known hypoxia-induced signaling pathways do appear to be playing a role. We and others have shown that the mitochondria play a crucial role in many hypoxic responses, including HIF-dependent transcriptional responses as well as many HIF-independent responses that don’t necessarily involve transcription at all [11–19, 42]. It is thought that the mitochondria, in response to the low oxygen levels increase their production of mtROS which signals the oxygen deprivation to the cellular machinery, eliciting various adaptive responses. While we did not directly measure mtROS production, inhibition of the mitochondrial electron transport chain with rotenone or myxothiazol, both of which have been shown to prevent hypoxic mtROS production and HIF-α stabilization [11, 12, 14, 15, 21, 44], prevented the ischemic stabilization of VEGF and MYC mRNAs suggesting that the mitochondria are playing a role. The fact that the glutathione peroxidase mimetic ebselen, which is known to reduce ROS levels in cells [11, 14], inhibited the response supports a role for mtROS in the hypoxic stabilization of mRNA that we observe.

Furthermore, our studies using antimycin A also implicate the generation of mtROS as a necessary step in the hypoxic stabilization of mRNA. Antimycin A inhibits mitochondrial respiration at the Qi site of Complex III, and has been shown to generate mtROS under normoxic conditions and mimic other known hypoxic responses [43–45]. What is so intriguing about our results is that antimycin A was able to induce mRNA stabilization in the presence of 1 g/L glucose but not 4 g/L glucose. This is in line with our previous studies showing that the hypoxic stabilization of mRNA was inhibited by 4 g/L glucose [25] and again supports a role for mtROS in this response. Taken together with the other inhibitor studies, these results provide strong evidence that mtROS production is both necessary and sufficient (in a low glucose environment) to induce the stabilization of mRNAs.

There are still a number of remaining questions regarding the hypoxic stabilization of mRNA. To date we have been unable to identify the RNA binding protein(s) and/or micro RNAs involved. Our thoughts going into this study is that we would hopefully find a HIF-induced target that we could pursue. However, it is clear from these results that it is unlikely to be a HIF-mediated transcriptional response. Instead, the involvement of mtROS suggests instead that this response is more likely to be regulated via post-translational modifications downstream of some signaling pathway. Previously, both P38 and Jun have been reported to be involved in the hypoxic stabilization of VEGF [37]. Furthermore, both have been found downstream of mtROS and have been implicated in other posttranscriptional responses including mRNA stability which makes them attractive candidates [23, 46, 49–52].

## Conclusions

Overall, this study demonstrates that hypoxic stabilization of mRNA occurs in a HIF-independent manner but requires the production of mtROS for proper execution of this adaptive response. Future studies will focus on identifying the downstream signaling pathways as well as the RNA binding proteins and/or microRNAs that mediate the posttranscriptional response. It is our hope that by better understanding all of the mechanisms by which hypoxia can alter gene expression we can develop improved therapeutics targeting cardiovascular disease, cancer and other diseases that involve periods of reduced oxygen.

## Abbreviations

ARE: AU Rich Element
ARNT: Aryl Hydrocarbon Receptor Nuclear Translocator
CoCl_2_: Cobalt chloride
DFX: Desferroxamine
DMOG: Dimethyloxalylglycine
HIF: Hypoxia Inducible Factor
HRE: Hypoxia Response Elements
mtROS: Mitochondrial reactive oxygen species
ODD: Oxygen dependent degradation domain
PHD: Prolyl hydroxylase
VHL: Von Hippel-Lindau
